# Supplementary material for: Robotic versus electromagnetic bronchoscopy for pulmonary leslon assessment using integrated intraprocedural imaging: Study protocol for the reliant 2 trial
Source: PLoS One. 2025 Dec 3;20(12):e0327611. doi: 10.1371/journal.pone.0327611 (PMC12674504; doi:10.1371/journal.pone.0327611)
Supplement: S1 File — (DOCX) [file pone.0327611.s001.docx]

**Title:** **R**obotic versus **E**lectromagnetic Bronchoscopy for Pulmonary **L**es**I**on **A**ssessme**NT** using integrated intraprocedural imaging: the RELIANT 2 trial.

**Principal Investigators:**

Fabien Maldonado, MD, MSc

Professor of Medicine and Thoracic Surgery

Professor of Mechanical Engineering

Vanderbilt University School of Medicine

Rafael Paez, MD, MSCI

Assistant Professor of Medicine

Division of Allergy, Pulmonary and Critical Care

Vanderbilt University Medical Center

| **Co-Investigators:** | Robert J. Lentz, MD  Jonathan D. Casey, MD, MSCI  Samira Shojaee, MD, MPH  Jennifer Duke, MD  Ankush Ratwani, MD  Greta Dahlberg, MD |
| --- | --- |
|  |  |
| **Biostatistician:** | Sheau-Chiann Chen, PhD  Heidi Chen, PhD |
|  |  |
| **Institutional Affiliations:**  **Version**  **Date** | Vanderbilt University Medical Center  Version 3.0  05/09/2025 |

Table of Contents

Study Synopsis 4

[Statement of Compliance](#_Toc50623983) 6

[Background and Significance](#_Toc50623983) 6

[Hypothesis and Study Objectives 7](#_Toc50623984)

[Methods](#_Toc50623986) 8

[General Study Design](#_Toc50623987) 8

[Study Population](#_Toc50623989) 8

[Endpoints](#_Toc50623987) 8

[Outcomes Definitions](#_Toc50623987) 10

[Recruitment and Enrollment Procedures 1](#_Toc50623988)1

[Randomization and Blinding 11](#_Toc50623993)

[Study Procedures 12](#_Toc50623993)

[Study Calendar 13](#_Toc50623996)

[Assesment of Resources 13](#_Toc50623995)

Informed Consent 14

[Data Collection 1](#_Toc50623990)6

[Statistical Considerations 1](#_Toc50624003)6

[Sample Size Calculation 1](#_Toc50623987)6

[Interim Analysis 1](#_Toc50623989)7

[Statistical Analysis Plan 1](#_Toc50623989)7

[Risks and Safety Reporting of Adverse Event 1](#_Toc50623999)9

[Reporting Period 20](#_Toc50623998)

[Procedure Related Risks 20](#_Toc50623998)

[Research Only Risks 20](#_Toc50623998)

Data Safety and Monitoring Board 21

[Regulatory Considerations 22](#_Toc50624004)

Privacy and [Confidentiality 22](#_Toc50624005)

[Protocol Review and Ammendments 22](#_Toc50624005)

[Good Clinical Practice 23](#_Toc50624005)

[Study Termination 23](#_Toc50624005)

[Benefit, Compensation and Additional Costs 23](#_Toc50624005)

[Trial Compliance 23](#_Toc50624005)

[Protocol Deviations 24](#_Toc50624005)

[Record Retention 24](#_Toc50624005)

[Plan for Dissemination of Findings 24](#_Toc50624003)

[References 25](#_Toc50624008)

**PROTOCOL SYNOPSIS**

| **Title** | **R**obotic versus **E**lectromagnetic Bronchoscopy for Pulmonary **L**es**I**on **A**ssessme**NT**: the RELIANT 2 trial |
| --- | --- |
| **Short title** | **RELIANT 2** Trial |
| **Primary study objective** | To compare the diagnostic yield of the Ion^TM^ Endoluminal System (robotic assisted bronchoscopy, RAB) with integrated cone beam computed tomography (CBCT) for intraprocedural imaging to that of ILLUMISITE™ Platform (electromagnetic navigational bronchoscopy, ENB) in patients undergoing bronchoscopy with planned biopsy of a pulmonary lesion. |
| **Study device** | Ion^TM^ Endoluminal System (RAB); ILLUMISITE™ Platform (ENB), OEC 3D GE HealthCare |
| **Design** | Multicenter, open label, pragmatic, superiority, cluster randomized controlled trial. |
| **Study centers** | Vanderbilt University Medical Center, Rush University Medical Center and Johns Hopkins University |
| **Sample size** | N=220 clusters, 440 subjects |
| **Inclusion  criteria** | 1. ≥ 18 years of age at time of bronchoscopy 2. Scheduled for navigational bronchoscopy for the evaluation of a pulmonary lesion |
| **Exclusion  criteria** | 1. Inability to provide informed consent 2. Enrolled in another study that requires the use of a specific platform |
| **Risks** | Participation in this study involves minimal risk because:   - All patients eligible for the study are already undergoing advanced diagnostic bronchoscopy for biopsy of a pulmonary lesion as part of routine clinical care. - Both RAB with integrated CBCT and ENB with integrated digital tomosynthesis are considered standard of care at VUMC and elsewhere. - Both RAB with integrated CBCT and ENB with integrated digital tomosynthesis are used interchangeably based on availability and provider preference. - If clinicians determine that RAB with integrated CBCT or ENB is required or contraindicated for the optimal care of an individual patient, the patient is excluded from the study. |
| **Consent** | Research informed consent to record and analyze participant data will be obtained at the time of the procedural clinical consent. |
| **Randomization** | Cluster randomization will be used for this study using operating room-days as clusters, given the impracticability of individual-level randomization. Clusters will be randomized to either RAB with integrated CBCT or ENB with integrated digital tomosynthesis. Randomization will be completed in permuted blocks of variable size stratified by room availability. |
| **Primary  outcome** | 1. The primary endpoint will be strict diagnostic yield defined as the proportion of procedures that result in acquisition of lesional tissue. Lesional tissue: histopathological findings present that readily explain the presence of a pulmonary nodule. |
| **Secondary outcomes** | 1. Duration of the procedure |
| **Exploratory endpoints** | 1. Need for additional diagnostic procedures 2. Radiation exposure 3. Best rEBUS signature 4. Diagnostic accuracy at 12-months post-biopsy 5. Suitability of specimens for molecular analysis |
| **Safety outcome** | Rate of complications (including pneumothorax, bronchopulmonary hemorrhage, respiratory failure and anesthesia complications) |
| **Follow-up** | Post procedure patients will be managed and followed per usual care. For patients not diagnosed with malignancy, we will review their interval chest CT scans to assess the target lesion for progression, regression, or stability for up to 12 months. |
| **Statistical  analysis** | The primary analysis will be a modified intention-to-treat comparison of patients randomized to RAB vs ENB with regard to the primary outcome of diagnostic yield. The study is designed to test the hypothesis that the diagnostic yield of RAB with integrated CBCT is superior to that of ENB with integrated digital tomosynthesis. |

**Statement of Compliance**

This human subject study will comply with all applicable federal, state, and local laws and regulations, including generally accepted standards of good clinical practice as adopted by current Food Drug Administration (“FDA”) regulations and statutes. The study site shall only allow individuals who are appropriately trained and qualified to assist in the conduct of the study.

**BACKGROUND AND SIGNIFICANCE**

Millions of nodules are detected every year in the United States. The majority are benign, but some represent early lung cancer and biopsy is needed to establish the diagnosis (1). Advanced imaging and navigational guidance systems are routinely used to sample these small peripheral lesions bronchoscopically (2, 3). A variety of navigational technologies are currently available, including electromagnetic navigational bronchoscopy (ENB) and robotic assisted bronchoscopy (RAB) (3,4), both cleared by the FDA via the 510(k) pathway. The latter is used clinically with either conventional fluoroscopy or with cone beam computed tomography (CBCT) guidance depending on provider preference.

Electromagnetic navigational bronchoscopy uses low frequency waves to track a sensor within an electromagnetic field to guide navigation to the lesion of interest (3). The largest prospective multicenter study assessing ENB diagnostic performance and safety reported a diagnostic yield of 67.8% and a complication rate of 4.7% (5). Digital tomosynthesis, which provides three-dimensional intraprocedural imaging and allows the operator to update the location of the lesion, has been integrated with ENB resulting to diagnostic yield estimates of 75-83% (6-8).

Since market release in 2019, few studies, mostly retrospective and observational studies have reported on the diagnostic yield of RAB which is estimated to be approximately 75-80% (9, 10). Most of these procedures were performed using conventional fluoroscopy which provides a two-dimensional image to assist with location of the bronchoscope within the chest and with biopsy. However, pulmonary nodules are frequently not visible with conventional fluoroscopy, particularly subsolid nodules, which may contribute to non-diagnostic procedures. Thus, the combination of RAB with CBCT, a three-dimensional cross-sectional imaging modality, has been widely adopted by the interventional pulmonology community (7-9). Cone beam CT produces a near real-time intraprocedural CT image that allows the proceduralist to manually reposition the robotic bronchoscope based on the location of the bronchoscope relative to that of the nodule. Preliminary data suggest that addition of CBCT improves the diagnostic yield (11-13). RAB is now integrated with CBCT, which allows the proceduralist to update the position of the nodule in the navigation system itself rather than manually. This upgrade is believed to increase the diagnostic yield of RAB.

We recently completed the RELIANT randomized controlled trial which compared the diagnostic yield of RAB to that of ENB. Results showed similar diagnostic yield (manuscript in preparation). While CBCT was used in approximately 50% of the cases at the proceduralist discretion, it was not integrated, and lesion update was not possible. Thus, we propose to compare the diagnostic yield of ENB with integrated digital tomosynthesis to that of RAB with integrated CBCT, as both bronchoscopy platforms are currently being used routinely and interchangeably at Vanderbilt University Medical Center (VUMC).

Both RAB with CBCT and ENB are performed in two operating rooms dedicated to interventional pulmonary procedures. Due to the set-up time required and patient workflow, each device is set up in one operating room and used for all patients undergoing biopsy in that room on that day. Patients are currently scheduled based on operating room availability without any consideration for the device that will be used. Similar to RELIANT (IRB# 221255), we leveraged this variation in clinical practice to design this pragmatic, randomized controlled study to test the hypothesis that the diagnostic yield of RAB with integrated CBCT is superior to ENB with integrated digital tomosynthesis in patients undergoing bronchoscopic biopsy of a peripheral pulmonary lesion.

**HYPOTHESIS AND STUDY OBJECTIVE(S)**

**Hypothesis**

In this study, we hypothesize that the use of RAB with integrated CBCT will result in higher diagnostic yield compared to ENB with integrated digital tomosynthesis in patients undergoing advanced diagnostic bronchoscopy to biopsy a pulmonary lesion.

**Objectives**

1. To test the hypothesis that the RAB with integrated intraprocedural CBCT will result in higher diagnostic yield compared to ENB in patients undergoing advanced diagnostic bronchoscopy to biopsy a pulmonary lesion.
2. To compare the rate of complications, procedure time, radiation exposure, diagnostic accuracy at 12 months, best radial endobronchial ultrasound (rEBUS) signature, specimen adequacy for molecular testing and need for additional procedures between these two bronchoscopy modalities.

**METHODS**

**General Study Design**

To evaluate our objectives, we propose a multicenter, open label, pragmatic, superiority, cluster randomized controlled trial. Patients undergoing advanced bronchoscopy to biopsy a pulmonary lesion will be assigned to either RAB with integrated CBCT or ENB based on cluster randomization. Allocation will be revealed each morning before procedures are started. Any advanced diagnostic bronchoscopy scheduled on that day will be performed with the platform (RAB with integrated CBCT or ENB) allocated to that room on that day. All decisions regarding procedure tools, techniques, and patient management will be per usual care and at the discretion of the treating physician.

**Study Population**

Inclusion Criteria:

1. ≥ 18 years of age at time of bronchoscopy
2. Scheduled for navigational bronchoscopy for biopsy of a pulmonary lesion

Exclusion Criteria

1. Inability to provide informed consent
2. Enrolled in another study that requires the use of a specific platform

**Endpoints**

Primary Outcome

1. The primary outcome will be strict diagnostic yield, defined as the proportion of procedures that result in acquisition of lesional tissue. Lesional tissue is defined by the presence of specific pathological findings that readily explain the presence of a pulmonary lesion (14). The following common pathological findings are pre-specified:

- Malignancy
- Specific benign pathologic findings including:
  - Organizing pneumonia
  - Frank purulence/robust neutrophilic inflammation
  - Granulomatous inflammation
  - Other specific benign findings such as hamartoma, amyloidoma or other uncommon causes of peripheral pulmonary lesions with distinctive pathological patterns as adjudicated by an expert lung pathologist.

Biopsies not meeting any of the above lesional pathological criteria will be adjudicated as not meeting the primary outcome (not being “diagnostic"), including biopsies with normal lung parenchyma or airway components on biopsy, atypia not diagnostic of malignancy, or non-specific inflammation. A blinded panel will review all non-malignant biopsies at the end of accrual to confirm primary outcome adjudication. Procedures will be adjudicated as not meeting the primary outcome if the procedure starts but biopsies are not obtained (due to failure to navigate to the lesion, complication, or equipment failure). A procedure will be considered started at first insertion of the bronchoscope.

Biopsies obtained without the use of guided bronchoscopy (e.g., sampling of central lymph nodes using the linear endobronchial ultrasound bronchoscope (EBUS) will not be included in the diagnostic yield calculations. Patients enrolled but diagnosis obtained on EBUS will be excluded as advanced diagnostic bronchoscopy would not be perform in these cases. Only the index bronchoscopy will be included in the diagnostic yield calculation (i.e. patients will only be enrolled once). Additional bronchoscopies, whether shortly after index bronchoscopy or later during the study period, will be recorded and analysed as an exploratory outcome (need for additional diagnostic procedures).

Secondary outcomes

1. Duration of bronchoscopy (in minutes), defined as time from beginning of registration steps to the removal of the catheter after completion of navigation procedures

Exploratory outcomes

1. Radiation exposure, defined as radiation dose delivered to the patient during the study bronchoscopy, recorded as a dose area product (mGy/cm2)
2. Need for additional diagnostic procedures directed at the lung nodule of interest
3. Diagnostic accuracy at 12-months post-biopsy
4. Best radial endobronchial ultrasound (rEBUS) signature at any point during procedure
5. Specimen suitability for molecular analysis when needed

Safety outcome

Safety outcomes will focus on procedural complications within 7 days of the procedure including clinically significant:

- Any pneumothorax
- Bronchopulmonary haemorrhage requiring additional intervention beyond suctioning, and wedging.
- Respiratory failure requiring any form of supplemental oxygen or respiratory support.
- Anesthesia complications

**Outcomes Definitions**

- *Rate of complications:* Number of procedures resulting in any complication divided by the total number of procedures.
- *Rate of specific complication:* Number of procedures resulting in a specific complication divided by the total number of procedures.
- *Need for additional diagnostic procedures:* Any diagnostic procedure performed after the study bronchoscopy which targets the same peripheral lesion (including repeat bronchoscopy, transthoracic needle biopsy, or surgical lung biopsy) will be considered an additional diagnostic procedure. Repeat biopsies of lesions determined to be malignant by study bronchoscopy which are 1) performed specifically to obtain additional tissue for further testing but that does not change the malignant diagnosis, or 2) therapeutic surgical resection of such lesions, will not be considered additional diagnostic procedures.
- *Diagnostic accuracy:* Number of true positive (malignant) lesions plus true negative (specific benign diagnosis) lesions with no evidence of malignancy at 12-month follow-up (no interval biopsy diagnostic of malignancy, regression on CT or stable size with no plan for repeat diagnostic procedure), divided by the total number of biopsied lesions.
- *Best rEBUS signature:* Concentric, eccentric or no rEBUS signature at any point during procedure
- *Specimen adequacy for molecular analysis when needed:* Proportion of specimens adequate for testing (quantity sufficient for testing) divided by the total number of specimens sent.

**Recruitment and Enrollment procedures**

All patients undergoing advanced diagnostic bronchoscopy for biopsy of a lung lesion will be screened for eligibility for this study based on the above eligibility criteria. Patients who do not meet inclusion criteria will be considered ‘ineligible.’ Patients who meet inclusion criteria but also meet at least one exclusion criterion will be considered ‘excluded.’ For patients who meet inclusion criteria but are not enrolled, the reason for exclusion will be recorded. The majority of advanced diagnostic bronchoscopy procedures at participating centers meet eligibility criteria for this study.

After confirming the patient meets eligibility criteria, the patient will be approached for enrollment in the study. Written research informed consent for the collection of data will be obtained at the time of procedural consent as done for our other randomized controlled trial (RELIANT IRB# 221255). Most patients undergoing bronchoscopy at VUMC are not seen in clinic before their bronchoscopy, and clinical consent for the procedure is obtained in the preoperative area immediately before the bronchoscopy. Given the broad inclusion criteria and comparison of two standard of care modalities commonly used in clinical practice, the number of patients expected to decline participation is small.

**Randomization and Blinding**

All patients meeting eligibility criteria for this study who sign the informed consent will be enrolled. Cluster randomization will be used for this study given impracticability of patient-level 1:1 parallel randomization (see Informed Consent section for details). All patients undergoing an advanced diagnostic bronchoscopy will have their procedure performed with the platform assigned to their operating room (OR) for the day. A biostatistician not involved in patient care will generate the randomization sequence. Random permuted blocks of size 2 and 4, stratified by OR availability will be used to ensure balanced allocation. A bronchoscopy scheduler with no knowledge of the allocation scheme will schedule patients for their procedures. Allocations will be concealed in sealed envelopes which will be opened every morning by the OR staff preparing the rooms.

It is not possible to blind the bronchoscopist or the patient to the platform used on the day of the procedure, as they are both large distinctive-appearing pieces of equipment that are operated differently. However, allocation will be concealed until the morning of the procedure so that bronchoscopists and bronchoscopy schedulers will remain blinded, and allocations will not influence scheduling of procedures in a given OR. Thoracic pathologists will remain blinded throughout the study.

**Study Procedures**

Written research informed consent for the collection of data will be obtained when obtaining procedural consent (refer to Informed Consent section below) and all eligible patients will be enrolled. An allocation envelope will be opened by the OR staff each morning to determine which platform (RAB with integrated CBCT or ENB) will be set up. The pre-operative, procedure, and post-operative steps will proceed per usual standard of care and as has been previously described (6, 9). The only aspect of care influenced by the study is which platform – RAB with integrated CBCT or ENB – will be set up in the room each day. Bronchoscopy will be performed by pulmonologists with expertise with both devices per standard of care. Procedures will be performed under general anesthesia with neuromuscular blockade per standard of care. Radial endobronchial ultrasound will be available for all procedures. CBCT spin will be performed per usual care at the discretion of the proceduralist and follow our routine protocol that includes positioning and collimation steps as well as a breath hold while spinning. Biopsies will be obtained using transbronchial needles, biopsy forceps, cytology brushes, cryoprobes, and/or other sampling devices at the discretion of the bronchoscopist. Rapid on-site evaluation will be performed to assess specimen adequacy. All patients will recover based on our usual standard of care, which includes two hours of monitoring in the PACU before being discharged. Imaging to assess for possible pneumothorax will be obtained if clinically indicated at the discretion of the proceduralist.

Post-procedure, patients will be managed and followed per usual care. For patients not diagnosed with malignancy, we will review their interval chest CT scans to assess the target lesion for progression, regression, or stability for up to 12 months.

**Study Calendar**

|  | **STUDY PERIOD** | | | | |
| --- | --- | --- | --- | --- | --- |
|  | **Enrolment** | **Allocation** | **Post-allocation** | | **Close-out** |
| **TIMEPOINT** | ***Pre-procedure (office or holding area)*** | **Procedure day** | ***Day 7*** | ***3-12***  ***months*** | ***12 months*** |
| **ENROLMENT:** | X |  |  |  |  |
| **Eligibility screen** | X |  |  |  |  |
| **Informed consent** | X |  |  |  |  |
| **Allocation** |  | X |  |  |  |
| **INTERVENTIONS:** |  | | | | |
| ***RAB + integrated CBCT*** |  | X |  |  |  |
| ***ENB*** |  | X |  |  |  |
| **ASSESSMENTS:** |  | | | | |
| ***Baseline and procedure data*** |  | X |  |  |  |
| ***Histopathology*** |  |  | X |  |  |
| ***Complications*** |  | X | X |  |  |
| ***Additional procedures*** |  |  |  | X |  |
| ***Diagnostic Accuracy*** |  |  |  |  | X |

**Assessment of Resource(s)**

The Interventional Pulmonology group at VUMC performs over 400 advanced diagnostic bronchoscopies per year and is one of the leading centers in navigational bronchoscopy in the US, both from a volume and expertise standpoint. The other 2 centers also have extensive expertise performing advanced bronchoscopies. The group at VUMC generally consists of 4 full time board-certified interventional pulmonologists, two advanced bronchoscopists, an interventional pulmonology fellow, a dedicated interventional pulmonology nurse practitioner, and a dedicated group of outpatient personnel (nurses, nurse navigators, and dedicated schedulers). Samples are reviewed per standard of care by a group of lung pathologists. Anesthesia for the procedures is provided by general and cardiothoracic anesthesiologists supervising CRNAs with specific expertise in bronchoscopy anesthesia.

**Informed Consent**

RAB with CBCT and ENB are routinely used to biopsy of pulmonary lesions. Both approaches represent usual care treatment and there are no comparative studies demonstrating the superiority of one approach over the other in clinical practice. The RELIANT trial (IRB# 221255), which is the only randomized controlled trial comparing RAB to ENB, showed that RAB is non-inferior ENB (manuscript in preparation). This trial will only enroll patients who are already scheduled to undergo advanced diagnostic bronchoscopy to biopsy a pulmonary lesion as part of routine clinical care for whom the proceduralist believes that either RAB with CBCT or ENB can be used.

Like RELIANT (IRB# 221255), a traditional 1:1 individual level randomization would be impracticable for this study. We currently perform advanced diagnostic bronchoscopy in two operating rooms within the main VUMC ORs, often simultaneously. Since we only have one ENB system and one RAB system, we often use them simultaneously in the two ORs; thus, individual level randomization would not be possible. Also, both systems require time to set up, including moving large pieces of equipment into and out the OR and running calibration steps. OR staff typically arrives one hour before procedures are scheduled to begin each day to set up. Each OR hosts 5-8 bronchoscopies daily, up to four of which on a given day may be ENB/RAB cases (often back-to-back with each other). Additionally, these procedures need to be planned by the bronchoscopist ahead of time. To accommodate traditional individual patient-level randomization would require moving these platforms between ORs multiple times daily and planning the procedure after consent and enrollment. This would result in a prohibitive disruption of our workflow, procedural delay, and suboptimal patient care rendering this study impracticable. Thus, we propose a *cluster randomized design*.

From the perspective of an individual patient planned to undergo bronchoscopic biopsy of a peripheral pulmonary lesion, it is currently arbitrary whether their biopsy will be performed with ENB or RAB, which depends on the day they happen to be scheduled and which of our two platforms (ENB and RAB) happen to be set up in the OR that day. Given that no comparative data exist showing superiority of one modality over the other, we have no clinical reason to prefer one system or the other. However, the provider retains autonomy to determine the most appropriate course of clinical care based on the presentation of the patient. If new information on treatment practices were to arise and one of our two platforms were recommended for specific cases, the corresponding patients would receive the appropriate treatment and would not be eligible for this study.

Participation in this study involves minimal incremental risk because:

- All patients eligible for the study are already undergoing bronchoscopic biopsy of a pulmonary lesion as part of routine clinical care.
- Bronchoscopic biopsy of a pulmonary nodule is currently done with either RAB with CBCT or ENB in routine clinical practice.
- Both RAB with CBCT or ENB are considered standard of care at VUMC and elsewhere in the US.
- RAB with CBCT or ENB are used interchangeably based on availability and provider preference.
- Benefits or risks are currently not known to differ between RAB with CBCT or ENB during bronchoscopic biopsy of a pulmonary lesion.
- If clinicians determine that RAB with CBCT or ENB is required or contraindicated for the optimal care of an individual patient, the patient is excluded from the study.

We believe the only feasible study design for this trial is cluster randomization as previously described. We recently finished enrollment for another cluster randomized controlled trial (RELIANT IRB# 221255) with a very similar design. As done for RELIANT and previously approved by the IRB, we will obtain research inform consent for the use of the participant data and to inform participants of the platform allocation (RAB with CBCT or ENB) as this will be pre-determined by randomization at the beginning of the day and before the patient arrives in the pre-operative area, where consent is most often obtained (only a minority of patients are seen in the office before the day of the procedure, and most patients are seen for the first time and clinically consented in the holding area immediately before the bronchoscopy). The procedure will be exactly the same whether patients agree to participate in the study or not.

The only risk of participation in this study beyond what is expected as part of routine care is a breach of patient confidentiality. All data collected as part of this study will be maintained in a secure REDCap database by proceduralists who have already been exposed to the patient’s PHI while providing clinical care. Research informed consent will be obtained when clinical informed consent is obtained for the procedure. Research informed consent will be scanned by a study member into the secured REDCap database and the research paper consent will be kept in a research binder in a locked file cabinet in a locked office. Only the study team will have access to the locked file cabinet. In addition, a note will be written in the patient’s electronic medical record stating that the patient was enrolled in this study.

**DATA COLLECTION**

All data will be collected and recorded in a Health Insurance Portability and Accountability Act compliant REDCap database (15). Data collected for the purposes of this study will come from two sources: [1] variables documented in the electronic health record as part of routine clinical care, [2] variables recorded by clinicians in REDCap. Data from the electronic medical record will be collected by trained study personnel (key study personnel) and recorded in the study’s REDCap database. The data collected for this study is very similar to the data collected as part of the RELIANT randomized controlled trial (IRB# 221255). As done for RELIANT, we will collect clinical data including but not limited to subjects’ demographic and clinical information, imaging information, follow up clinic visits, interval imaging, PET, invasive procedures, procedure complications, and pathology results for up to 12 months. Data access groups have been created within REDCap so that no PHI will be shared or seen across sites. Only De-identified data will be shared at the end of the study by external sites. There is no current plan to share individual VUMC data with external sites.

**Statistical Considerations**

**Sample Size Estimation**

The diagnostic yield of ENB varies widely in the literature (2). Based on data from prior studies and our own published data, we estimated the diagnostic yield of ENB to be 80% (6, 9). There is very limited data on the diagnostic yield of RAB with integrated CBCT (11-13); however, a 10% increment in diagnostic yield would be considered clinically significant and thus influence practice. Assuming the diagnostic yield of ENB is 80% and cluster size of 2, 110 clusters (OR-days) per group will provide at least 80% power to detect a 10% increment in diagnostic yield (i.e. 90% diagnostic yield in RAB with integrated CBCT) at a two-sided type I error rate of 5%, with intracluster correlation of 0.1 (based on prior data from RELIANT). Based on an average of 2 subjects per cluster, we will enroll 440 participants for the trial.

**Interim Analysis**

This trial will be monitored for efficacy at 50% enrollment (220 evaluable participants) and at the end of the study. The Z statistics of efficacy boundary at first interim analysis and at the end of study are 2.96 and 1.97 using O’brien-Fleming spending functions at a one-sided type I error rate of 0.025. The efficacy stopping threshold for the first interim and final analysis as inferred from the boundary to be p<0.0015 and p< 0.025 respectively.

**Statistical Analysis Plan**

*Descriptive Analysis*

To characterize the study sample, baseline demographic and clinical data will be described overall and by group. Categorical variables will be described using frequencies and proportions, and continuous variables will be described using means and standard deviations, as well as medians and interquartile ranges. Missingness will be reported for each variable.

We will describe the outcome variables overall and grouped by study arm using the same approach as for the demographic data. Summary statistics and graphical representations may be displayed, and missingness will be reported for each variable. No statistical comparisons between groups will be made for this descriptive analysis.

*Primary outcome*

The primary analysis for the trial will use a modified intent-to-treat approach as patient who do not undergo navigation bronchoscopy because diagnosis is obtained on EBUS will be excluded from the analysis as described in prior sections. Participants will be evaluated by treatment group as assigned regardless of what was delivered. All eligible participants will be included.

The primary outcome variable (diagnostic yield) will be compared between groups using a generalized linear mixed model with two-sided test. The primary model will be covariate adjusted, including fixed effects for device assignment, lesion size, density, peripheral location, bronchus sign, and operator. Should the model demonstrate signs of overfitting, covariates may be selected based on priority order (device assignment, lesion size, density, peripheral location, bronchus sign, operator).

*Secondary and exploratory outcome*

A sensitivity analysis using a *per protocol* approach will be conducted to analyze participants based on the device used. We will use the same approach as the primary analysis, an adjusted generalized linear mixed effects model using the same covariates.

The secondary outcome, procedure duration, will be compared between study groups using a linear mixed model. If the procedure duration is skewed, alternative models may be pursued, such as a cox regression. Analysis of the exploratory endpoints will follow a similar approach.

*Safety analysis*

Procedure complications are expected in usual care, although uncommon. We will report all procedure complications for each device, overall and by type. If event rates exceed 5%, we may proceed with a comparative analysis, which will involve a generalized linear regression model for binary outcomes as specified for the main analysis, with the exception that covariate adjustment may not be possible. The safety analysis dataset will group participants by device used, regardless of assignment.

All model results will be summarized with point estimates and 95% confidence intervals (CIs), which will be emphasized over p-values when reporting the results for secondary outcomes. No adjustments for multiplicity will be made.

*Differential effects*

To determine whether differences in outcomes are dependent on baseline characteristics, we will introduce interaction terms into the models developed for the main analysis. Specifically, we will test the interaction between device assignment and the subgrouping variable. Each variable will be tested one by one, such that all main effects but only one interaction term is included at a time by the likelihood-ratio test. The following putative subgrouping variables are prespecified:

- Nodule size (continuous and grouped as <1.5cm, 1.5-3cm, >3cm)
- Presence of bronchus sign
- Solid vs subsolid nodule
- Peripheral vs central location – *Peripheral defined as outer 1/3 of chest* *as assessed visually by the proceduralist (as in RELIANT).*

Missingness on the primary or secondary outcome is not expected due to the proximity of its measurement with the procedure and its integration into clinical documentation. Procedures missing the primary outcomes will be considered not diagnostic. Missing covariates will be imputed using multiple imputation with predictive mean matching. There may be missingness on exploratory outcomes. For missing exploratory outcomes, a complete case analysis will be performed.

**Risks and Safety Reporting of Adverse events**

We believe this pragmatic randomized controlled trial to be of minimal incremental risk as discussed above. Advanced diagnostic bronchoscopic to biopsy of a pulmonary lesion is routinely done with either RAB with CBCT or ENB in routine clinical practice. Furthermore, the proceduralist remains in complete control e.g., no aspect of the procedure is dictated by the study, and if RAB with CBCT or ENB is indicated/contraindicated the patient is excluded from the study. Information on adverse events, whether serious or not, whether reported by the participant, directly observed, or detected by physical examination, laboratory test or other means, will be collected, recorded, followed, and reported as described in the following sections.

**Reporting Period**

This pragmatic randomized controlled trial is embedded within usual clinical care and compares two standard of care approaches to biopsy peripheral pulmonary lesions. Clinical adverse events are well documented in the literature and discussed in detail with the patient during the clinical procedure consent. Clinical adverse events are, therefore, considered to be related to the clinical procedure rather than related to the research. Clinical adverse events are systematically collected and reviewed by the DSMB but not individually reported to the IRB. The primary risk of research participation is breach in privacy and confidentiality and many safeguards have been established as detailed previously. Serious unanticipated adverse events will be reported to the DSMB and IRB per current institutional standards.

- Any serious or non-serious adverse event related to research procedures (i.e., the consent process, HIPAA compliance, etc.) will be collected.
- Any serious unanticipated adverse event that occurs ≤ 7 days after the procedure.
- A written report will be sent to the DSMB and IRB within 7 calendar days of the PI being notified.

**Procedure Related Risks**

RAB with CBCT or ENB are considered standard of care and are being undertaken for routine care of the patient undergoing biopsy of a pulmonary lesion. As such, we do not expect that enrollment in this study will result in an increased risk for the patient above what they are experiencing as part of their routine care. The risks of navigational bronchoscopy for the biopsy of pulmonary lesion are well defined, and include pneumothorax, bronchopulmonary hemorrhage, respiratory failure, infection, and anesthetic complications. Risk of death from bronchoscopy is estimated around 1/20,000. Pneumothorax is the most common complication, occurring in approximately 2-5% of cases (9, 16) and may result in the need for a chest tube. Procedural risks are collected in the CRF and may be reviewed by the DSMB. As these are considered procedural risks and not research-related risks, they will not be reported to the IRB.

**Research Only Risks**

Key additional risks for study participants are data protection. All patient related information in this study will be entered and stored at Vanderbilt University Medical Center REDCap database, which requires two factor authentications if accessed from outside of VUMC’s firewall. Only relevant key study personnel approved by the IRB will have access to this database as necessary to conduct the research. Every effort will be made to protect the privacy of research subjects. Subject names and protected health information (PHI) will be kept confidential to the extent possible and as required by applicable laws and regulations. All records and data related to the study will be maintained in secure protected spaces, with access restricted to key study personnel approved by the IRB who (i) need access to the information to fulfill the terms and obligations under the Protocol and (ii) are under the same obligations as study personnel to keep the information confidential. Data access groups have been created within REDCap so that no PHI will be shared or seen across participating sites. Only de-identified data will be shared at the end of the study by external sites. There is no current plan to share individual VUMC data with external sites.

**DATA AND SAFETY MONITORING BOARD (DSMB)**

The principal role of the DSMB is to assure the safety of patients in the trial. They will regularly monitor safety data from this trial, review and assess the performance of its operations, and make recommendations to the study team with respect to:

- Participant safety and risk/benefit ratio of study procedures and interventions
- Protocol amendments (with specific attention to study population, intervention, and study procedures)
- Adherence to the protocol requirements
- Possible early termination of the trial because of new external information, early attainment of study objectives, safety concerns, or inadequate performance

The DSMB will be asked to evaluate any SAEs or unanticipated AEs. Outcome data may be presented to the DSMB at the DSMB’s request and during the planned interim analysis at 50% enrollment. The DSMB will consist of members with expertise appropriate to the conduct of the study, such as pulmonary medicine, biostatistics, and clinical trials. Appointment of all members is contingent upon the absence of any conflicts of interest. All the members of the DSMB are voting members. The Principal Investigators and unblinded study biostatistician will be responsible for the preparation of all DSMB and adverse event reports. The DSMB will develop a charter and review the protocol during its first meeting. Subsequent DSMB meetings will be scheduled in accordance with the DSMB Charter with the assistance of the Principal Investigator. The DSMB will have the ability to recommend that the trial end, be modified, or continued unchanged.

**Regulatory CONSIDERATIONS**

**Privacy and Confidentiality**

All patients will be assigned a unique study ID number for use in the coded study database. Only key study personnel will access patients’ electronic health records. This may occur at multiple timepoints during the study period including immediately following enrollment; when collecting baseline demographics and comorbidities (may occur anytime between enrollment and final data collection); and when collecting follow up data and clinical outcomes up to 1 year. The electronic health record may be accessed again, as needed, between enrollment and study publication to respond to queries focused on ensuring data completeness and quality. The minimal PHI that is collected will be visible only to site investigators at the site where the patient was enrolled. The dataset for analysis will contain the unique study ID and no other patient identifiers. At the time of publication, a fully de-identified version of the database will be generated.

At no time during this study, its analysis, or its publication will patient identities be revealed in any manner. The minimum necessary data containing patient or provider identities will be collected. Data collected from the medical record will be entered into the secure online database REDCap. Following publication of the study results, any hard copies of data collection forms will be destroyed, and the REDCap database will be fully de-identified in accordance with institutional regulations.

**Protocol Review and Amendments**

Information regarding study conduct and progress will be reported to the Institutional Review Board (IRB) per current institutional standards. The trial will not be initiated until there is approval by the IRB of the protocol. The IRB should be duly constituted according to regulatory requirements. The investigator will inform the IRB of the progress of the trial at least yearly. Any changes to the protocol will be made in the form of a written amendment and must be approved by the IRB prior to implementation. Protocol changes to eliminate an immediate hazard to a trial patient may be implemented by the investigator immediately. The investigator must then immediately inform the IRB and DSMB.

**Good Clinical Practice**

This study will be carried out in compliance with the protocol and Good Clinical Practice (GCP), as described within:

1. ICH Harmonized Tripartite Guidelines for Good Clinical Practice 1996.
2. Declaration of Helsinki, concerning medical research in humans (Recommendations Guiding Physicians in Biomedical Research Involving Human Subjects, Helsinki 1964, amended Tokyo 1975, Venice 1983, Hong Kong 1989, Somerset West 1996).

The investigator agrees to adhere to the instructions and procedures described within the above and thereby to adhere to the principles of Good Clinical Practice with which the above conform.

**Study Termination**

Reasons for study termination may include, but are not limited to, the following:

1. Investigator non-compliance with the protocol, GCP or regulatory requirements

2. Insufficient enrollment

3. Safety concerns

4. Decision by suppliers to modify or discontinue the availability, development or manufacture of protocol-indicated treatment or device

5. A request to discontinue the study by the IRB or a recognized regulatory authority

## **Benefits, Compensation and Additional Costs**

There will be no financial compensation for participation. There is no additional benefit to the patient by participating in the trial. Data from this study will be beneficial to the field.

There will be no additional cost to subjects for participating in this study. Subjects and/or their insurance companies will be responsible for all care provided as part of the procedure as this service is part of the standard of care they would receive for their condition.

**Trial Compliance**

This is an investigator-initiated study. The Principal Investigators, Fabien Maldonado, M.D and Rafael Paez M.D. are conducting the study and Vanderbilt University Medical Center (VUMC) will act as the sponsor.

**Protocol Deviations**

Vanderbilt University Medical Center is responsible for implementing and maintaining quality assurance and quality control to ensure that studies are conducted according to the protocol, GCP, and all applicable regulatory requirements. A protocol deviation is any noncompliance with the protocol. Noncompliance can be on the part of the study participant, the investigator, or the study site staff. Deviations to the protocol are not permitted except when necessary to eliminate an immediate hazard to study subjects.

**Record Retention**

An electronic case report form (eCRF) is required and must be completed for each included participant. Records will be retained compliant with institutional, federal, and local regulations. Secondary use of the data will be with IRB approval. The dataset may be made available outside of the study team on reasonable request with approval from an authorized Institutional Review Board and concurrence with the study team that the data are fit for purpose.

**PLANS FOR DISSEMINATION OF FINDINGS**

Any manuscript or releases resulting from the collaborative research must be approved by the investigator and will be circulated to applicable participating investigators prior to submission for publication or presentation. A publication plan consistent with the international Committee of Medical Journal Editors (ICMJE) will be created prior to analysis and publication of any data. All data will be made available to authors as required. The publication of sub-studies and post-hoc analyses will not precede the primary publication. Publication of results will be determined by the investigators. All authors are expected to disclose financial or affiliations that could be considered conflicts of interest per journal or medical society requirements.

**REFERENCES**

1. Gould MK, Tang T, Liu IL, Lee J, Zheng C, Danforth KN, et al. Recent Trends in the Identification of Incidental Pulmonary Nodules. Am J Respir Crit Care Med. 2015;192(10):1208-14.
2. Nadig TR, Thomas N, Nietert PJ, Lozier J, Tanner NT, Wang Memoli JS, et al. Guided Bronchoscopy for the Evaluation of Pulmonary Lesions: An Updated Meta-analysis. Chest. 2023;163(6):1589-98.
3. Criner GJ, Eberhardt R, Fernandez-Bussy S, Gompelmann D, Maldonado F, Patel N, et al. Interventional Bronchoscopy. Am J Respir Crit Care Med. 2020;202(1):29-50.
4. Agrawal A, Hogarth DK, Murgu S. Robotic bronchoscopy for pulmonary lesions: a review of existing technologies and clinical data. J Thorac Dis. 2020;12(6):3279-86.
5. Folch EE, Bowling MR, Pritchett MA, et al. NAVIGATE 24-Month Results: Electromagnetic Navigation Bronchoscopy for Pulmonary Lesions at 37 Centers in Europe and the United States. *J Thorac Oncol*. 2022;17(4):519-531.
6. Katsis J, Roller L, Aboudara M, Pannu J, Chen H, Johnson J, et al. Diagnostic Yield of Digital Tomosynthesis-assisted Navigational Bronchoscopy for Indeterminate Lung Nodules. J Bronchology Interv Pulmonol. 2021;28(4):255-61.
7. Aboudara M, Roller L, Rickman O, Lentz RJ, Pannu J, Chen H, et al. Improved diagnostic yield for lung nodules with digital tomosynthesis-corrected navigational bronchoscopy: Initial experience with a novel adjunct. Respirology. 2020;25(2):206-13.
8. Avasarala SK, Roller L, Katsis J, Chen H, Lentz RJ, Rickman OB, et al. Sight Unseen: Diagnostic Yield and Safety Outcomes of a Novel Multimodality Navigation Bronchoscopy Platform with Real-Time Target Acquisition. Respiration. 2021:1-8.
9. Low SW, Lentz RJ, Chen H, Katsis J, Aboudara MC, Whatley S, et al. Shape-Sensing Robotic-Assisted Bronchoscopy vs Digital Tomosynthesis-Corrected Electromagnetic Navigation Bronchoscopy: A Comparative Cohort Study of Diagnostic Performance. Chest. 2023;163(4):977-84.
10. Kalchiem-Dekel O, Connolly JG, Lin IH, Husta BC, Adusumilli PS, Beattie JA, et al. Shape-Sensing Robotic-Assisted Bronchoscopy in the Diagnosis of Pulmonary Parenchymal Lesions. Chest. 2021.
11. Abia-Trujillo D, Folch EE, Yu Lee-Mateus A, Balasubramanian P, Kheir F, Keyes CM, Villalobos R, Chadha RM, Hazelett BN, Fernandez-Bussy S. Mobile cone-beam computed tomography complementing shape-sensing robotic-assisted bronchoscopy in the small pulmonary nodule sampling: A multicentre experience. Respirology. 2023 Nov 28. doi: 10.1111/resp.14626. Epub ahead of print.
12. Styrvoky K, Schwalk A, Pham D, Madsen K, Chiu HT, Abu-Hijleh M. Radiation dose of cone beam CT combined with shape sensing robotic assisted bronchoscopy for the evaluation of pulmonary lesions: an observational single center study. J Thorac Dis. 2023 Sep 28;15(9):4836-4848.
13. Reisenauer J, Duke JD, Kern R, Fernandez-Bussy S, Edell E. Combining Shape-Sensing Robotic Bronchoscopy With Mobile Three-Dimensional Imaging to Verify Tool-in-Lesion and Overcome Divergence: A Pilot Study. Mayo Clin Proc Innov Qual Outcomes. 2022 Apr 23;6(3):177-185.
14. Gonzalez AV, Silvestri GA, Korevaar DA, et al. Assessment of Advanced Diagnostic Bronchoscopy Outcomes for Peripheral Lung Lesions: A Delphi Consensus Definition of Diagnostic Yield and Recommendations for Patient-centered Study Designs. An Official American Thoracic Society/American College of Chest Physicians Research Statement. *Am J Respir Crit Care Med*. 2024;209(6):634-646. doi:10.1164/rccm.202401-0192ST
15. Harris PA, Taylor R, Thielke R, Payne J, Gonzalez N, Conde JG. Research electronic data capture (REDCap)--a metadata-driven methodology and workflow process for providing translational research informatics support. J Biomed Inform. 2009;42(2):377-81.
16. Ost DE, Ernst A, Lei X, Kovitz KL, Benzaquen S, Diaz-Mendoza J, et al. Diagnostic Yield and Complications of Bronchoscopy for Peripheral Lung Lesions. Results of the AQuIRE Registry. Am J Respir Crit Care Med. 2016;193(1):68-77.
